# *Typhlomystaxuansis* (Rodentia, Platacanthomyidae): new species of the genus from northern Vietnam with notes on conservation status and distribution

**DOI:** 10.3897/BDJ.12.e133363

**Published:** 2024-10-29

**Authors:** Alexander E Balakirev, Bui Xuan Phuong, Viatcheslav V Rozhnov

**Affiliations:** 1 A.N.Severtsov's Institute of Ecology and Evolution of Russian Academy of Sciences, Moscow, Russia A.N.Severtsov's Institute of Ecology and Evolution of Russian Academy of Sciences Moscow Russia; 2 Joint Vietnam-Russia Tropical Science and Technology Research Centre, Hanoi, Vietnam Joint Vietnam-Russia Tropical Science and Technology Research Centre Hanoi Vietnam

**Keywords:** Southeast Asia, Vietnam, rodents, taxonomy, biodiversity

## Abstract

**Background:**

The paper presents novel findings of little-known species of rodents, the blind tree mice *Typhlomys* in Son La Province, Vietnam, with the first morphological and genetic characterisation and taxonomical description of the new species, *T.taxuansis*. The study also summarises all the data available on this genus species distribution, museum collections and notes on its taxonomy, which are important to establish the proper conservation status of the species. An exhaustive map of the findings is provided, along with a refined taxonomic key for all six currently morphologically characterised species of the genus. It is shown that, based on the data available to date, the genus is still far from complete. Most species, apparently, do not need a special conservation measure; their status may be established as Least Concerns and Near Threatened (B1a+2a) and the current population trend is stable (IUCN).

**New information:**

The paper introduced innovative findings regarding lesser-known rodents, the blind tree mice *Typhlomys* in Son La Province, Vietnam, along with the primary morphological and genetic identification and taxonomic explanation of the novel species *T.taxuansis*.

## Introduction

Platacanthomyidae is a relict rodent family (Musser and Carleton 2005) represented by only two recent genera with 6-7 species (Abramov et al. 2014). For a long time, it was considered an enigmatic family due to the fact that its external morphologies are much like those of dormice (Gliridae), the anatomical structures of the bullae and dentary are more similar to muroids (Miller and Gidley 1918, Musser and Carleton 2005) and their molar occlusal pattern shares a considerable part of features with the Crecitidae (Fejfar and Kalthoff 1999) and Nesomyinae (Ellerman 1940, Ellerman 1949). Their evolutionary relationships have remained uncertain for a long time (Hong 1982) until molecular evidence supported Platacanthomyids as a distinct lineage that composes the most basal clade of Muroidea families (Jansa et al. 2009). Thus, the English common name “pigmy dormice” (Wilson and Cole 2000) as well as the Russian one “Chinese dormouse hamsters” (Sokolov 1984) in this case are obviously misleading. In the latest taxonomic summary of mammals, they are assigned the common name “tree mice” (Giarla 2017), which should be followed today. Taking into consideration its most specific feature, the most common would be the name “blind tree mice” to distinguish it from other groups of arboreal mice.

The type species of the genus, *Typhlomyscinereus*, was described from Fujian, China (Milne-Edwards 1877) and is now known over a fairly large range in central China (Hong 1982, Wang et al. 1996, Liu et al. 2007, Smith 2008, Cheng et al. 2017). Based on differences in body size and fur colouration, several additional taxonomic entities have been described more recently (Wang et al. 1996, Musser and Carleton 2005) along with a number of related taxa known from isolated highland populations in southern and central China and the Hoang Lien Range in northern Vietnam (Liang 1980, Liu et al. 1984, Liu et al. 2007, Can et al. 2008, Abramov et al. 2012, Abramov et al. 2014). According to a review by Wang et al. (1996), the nominate subspecies *T.c.cinereus* was found to be distributed in northern Fujian and Zhejiang Provinces and southern Anhui and has also recently been recorded in Guangdong Province, China (Cong et al. 2013). Three other Chinese taxa have restricted ranges. *T.c.daloushanensis* Wang et Li, 1996, is known from southern Sichuan, Shaanxi, Gansu, Hubei and Guizhou; *T.c.guangxiensis* Wang et Chen, 1996, is distributed in the southwest of Guangxi; and *T.c.jingdongensis* Wu et Wang, 1984, was discovered in Yunnan.

**Figure 1.**
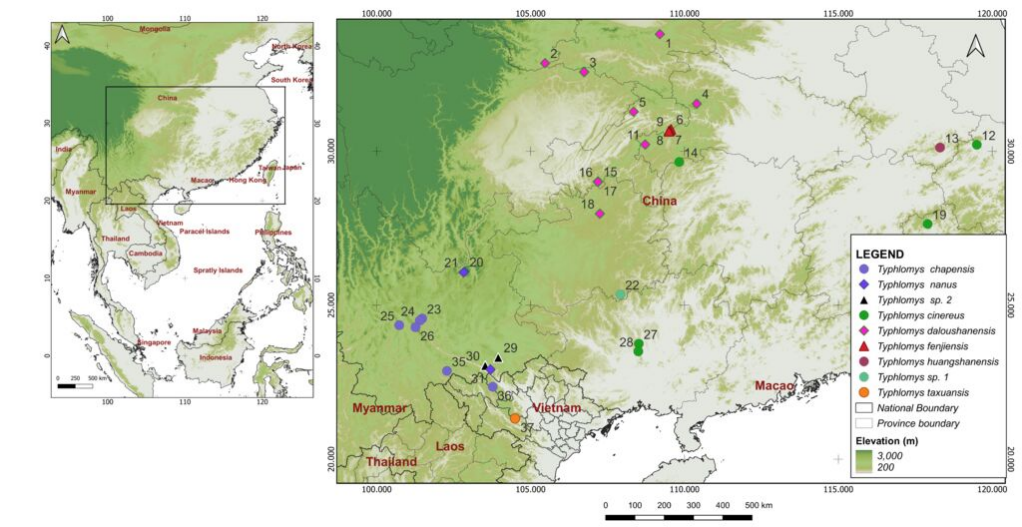
Distribution map for genetically investigated *Typhlomys* species and lineages.

The Vietnamese population was initially described by Osgood (1932) as a separate species, *T.chapensis*, which had long been considered a subspecies of *T.cinereus* (Corbet and Hill 1991, Wang et al. 1996, Musser and Carleton 2005) until its taxonomic status was restored (Abramov et al. 2014). A recent paper (Cheng et al. 2017) carried out the first successful revision of the genus, based on a combination of morphological and molecular genetic data. The study demonstrated that *T.chapensis* and *T.c.jingdongensis* are indistinguishable from each other. *Typhlomysc.cinereus* and *T.c.daloushanensis* also show the closest resemblance in dental morphology, but exhibit considerable genetic distances between samples. In a subsequent series of surveys (Hu et al. 2021, Pu et al. 2022), another species was described, along with two more genetic lineages of species rank that still do not have a formal description based on morphological materials. Thus, under the most recent view, the genus comprises five established species, namely *cinereus*, *chapensis*, *daloushanensis*, *fengjiensis* and *nanus*. There are also another two putative species, usually called *Typhlomys* sp. 1 and *Typhlomys* sp. 2 (Hu et al. 2021), still remaining morphologically unverified (Fig. 1).

In spite of these findings, the composition of the genus still cannot be considered completely clear due to the scarcity of museum materials available for study and the obvious fragmentation and disjunctive pattern of the natural area of the genus. Thus, in particular, the southern limit of the distribution of the genus in Indochina and the species composition in Vietnam remain debatable. Little is known about the natural history of these rodents because only a few scientists have been lucky enough to observe them in nature, in their natural habitats in high mountain cloud forests.

## Materials and methods

The small terrestrial mammals were trapped by snap traps during one of the recent theriological expeditions organised by the Russian-Vietnamese Tropical Research and Technological Center in Son La Province, Bac Yen District, within Xin Vang, Hang Dong, Ta Xua, Bac Yen, Song Pe, Hong Ngai, Ta Khoa and Hua Nhan communes, within and in the vicinity of Ta Xua Nature Reserve. The field surveys were carried out during the period of 21-30 November 2023.

One individual of *Typhlomys* (adult female) was obtained on 25 November 2023, in a moist forest in Ta Xua Nature Reserve, 4 km east of Y Xoa Homestay (21,32218 N, 104.495873 E, about 2100 m a.s.l., Fig. 1, point 37). It was used to obtain both morphological (skull and skin) and genetic samples. The whole body of the animal was initially preserved in 70% ethanol, followed by the skull and skin, which were processed for deposit in the Zoological Museum of Moscow State University, Moscow (ZMMU S-210284).

Direct measurements were taken in field and then a set of twenty-three cranial traits were analysed in the laboratory post-skull extraction and boiling. Final skull treatments have been made by larvae of *Dermestes* sp. obtained from the ZMMU collection, followed by cranial characters taken using digital calipers to the nearest 0.01 mm: Head and body length (LHB), tail length (LT), hind foot length (LHF), length of ear loop (LE), body weight (BW), occipitonasal length (ONL), zygomatic breadth (ZB), interorbital breadth (IB), length of rostrum (LR), breadth of rostrum (BR), breadth of braincase (BBC), height of braincase (HBC), breadth of zygomatic plate (BZP), length of diastema (LD), length of incisive foramina (LIF), breadth of incisive foramina (BIF), length of bony palate (LBP) (palatal bridge), breadth across bony palate at first molars (BBP), postpalatal length (PPL), breadth of mesopterygoid fossa (BMF), length of bulla (LB), crown length of maxillary molar row (CLM^1-3^), breadth of first upper molar (BM^1^) crown length of mandibular molar row (CLM_1-3_), breadth of first lower molar (BM_1_) following Musser and Newcomb (1983) and Musser et al. (2006).

For comparison, we summarised all data on *Typhlomys* with genetic species attribution and geographical locality availability, including our current and previously published data (Abramov et al. 2012, Abramov et al. 2014). The total dataset combines 37 points and 74 individuals from Vietnam and China, with 63 individuals attributed to the exact museum voucher or sample (Table 1). We follow the dental nomenclature system of molars proposed by Qiu (1989), who compared both recent and fossil species of *Typhlomys*.

For genetic analyses, small pieces of liver were stored in 96% molecular-grade ethanol and used for DNA extraction. The total genomic DNA was extracted using a routine phenol/chloroform/proteinase K protocol (Kocher et al. 1989, Sambrook et al. 1989). The individual has been genotyped by partial *Cyt b* (398–1140 bp positions), the *COI* gene (680 bp) and the growth hormone receptor partial sequence gene (*GHR*, 815 bp) and analysed together with all homologous sequences available in the GenBank (Abramov et al. 2014, Cheng et al. 2017, Hu et al. 2021, Pu et al. 2022). Universal routine PCR protocols have been used to amplify mtDNA fragments as follows: initial denaturation for 1 min 30 sec at 95°C, denaturation for 30 sec at 95°C, annealing for 1 min at 52°C and elongation for 45 sec at 72°C, followed by terminal elongation for 3 min at 72°C. The PCR reaction was performed in a 25µl volume that contained 2.5–3 ml of 10x standard PCR buffer (Fermentas), 50 mM of each dNTP, 2 mM of MgCl_2_, 10 pmol of each primer, 1 unit of Taq DNA polymerase (Fermentas) and 20–50 ng of total DNA template per tube. The *Cyt b* gene was amplified directly using primer pairs L14724 (Irwin et al. (1991), 5'-CGAAGCTTGATATGAAAAACCTCGTTG-3') and H15915, 5’-GGAATTCATCTCTCCGGTTTACAAGAC-3' (Kocher et al. 1989). For the COI gene, we sequenced the one-time routine BOLD primers LCO1490 and HCO2198 (5'-GGTCAACAAATCATAAAGATATTGG-3' and 5'-TAAACTTCAGGGTGACCAAAAAATCA-3') and protocols have been used as explained in Hebert et al. (2003). The GHR gene was amplified by the two-round nested scheme explained in Jansa et al. (2009) with primers GHRF1, 5’-GGRAARTTRGAGGAGGRGAACACMATCTT; GHRF50, 5’-TTCTAYARYGATGACTCYTGGGT-3'; GHR930R, 5’-RTAGCCACANGANGAGAGRAA-3'; and GHRendAlt, 5’-GATTTTGTTCAGTTGGTCTGTGCTCAC. The double-stranded DNA products were directly sequenced in both directions using an ABI PRISM 3730xl Genetic Analyzer (Applied Biosystems, USA) and the BigDye® Terminator v.3.1 Cycle Sequencing Kit (Life Technologies Corporation, Carlsbad, CA, USA) in agreement with the manufacturer’s protocol. Sequences obtained were deposited to GenBank database under IDs PP987021-PP987022 and PP987159.

A number of external GenBank deposited sequences (KX778415-KX778416, KC209551-KC209552, KX778366-KX778385, KX778340-KX778360, KX778394-KX778414, KX778361-KX778362; MT219901-MT219904, MT232968-MT232971; OL753459-OL753468, OL693252-OL693261, OL753439-OL753448, OL691088- OL691090) were used as a united dataset. Therefore, this dataset combines all the bulk of genetic information currently available for this genus for all specific populations currently discovered. We also used a number of outgroups exactly as in the paper Hu et al. (2021), namely *Jaculusjaculus* (KM397186, AJ416890, KM397231), *Myospalaxaspalax* (AF326272, KP724691, GQ272599) and *Rattusrattus* (HM217733, EU273707, AM910976) for full integrity.

Sequencing analyses, based on a concatenated 3643-bp-long sequence and individual *Cyt b*, *COI* and *GHR* genes, were conducted in MEGA X (Kumar et al. 2018). The phylogeny was inferred by the Maximum Likelihood Method and the General Time Reversible Model (Nei and Kumar 2000) as they are the most complex, universal and need no initial presumption about the codon evolution mode. The tree with the highest log likelihood (-5938.78) was used. The initial tree for the heuristic search was obtained automatically by applying the Neighbour-Joining and BioNJ algorithms to a matrix of pairwise distances estimated using the Maximum Composite Likelihood (MCL) approach and then selecting the topology with a superior log-likelihood value. A discrete Gamma distribution was used to model evolutionary rate differences amongst sites (5 categories +G parameter = 1.235). The rate variation model allowed for some sites to be evolutionarily invariable ([+I], 45.564% sites). All positions with less than 95% site coverage were eliminated, i.e. fewer than 5% alignment gaps, missing data and ambiguous bases were allowed at any position (partial deletion option), resulting in a total of 1943 positions in the final dataset. Bootstrap values were calculated with 10,000 iterations. Estimates of evolutionary divergence within *Typhlomys* species have been made by the *Cyt b* gene, as Nei and Kumar (2000).

### Results

**Figure 2.**
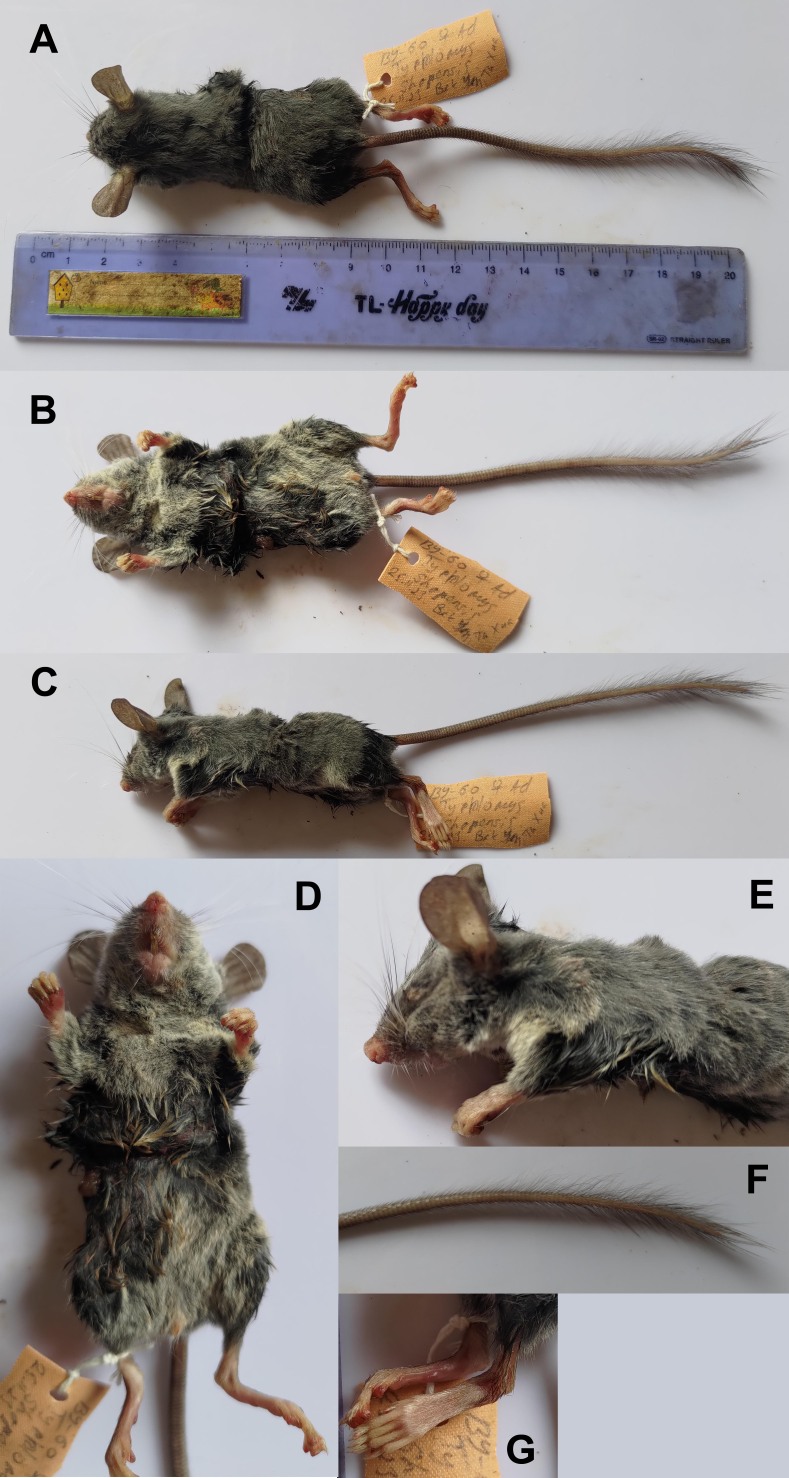
A new species of *Typhlomys* from Ta Xua Nature Reserve, specimen BY-60, adult female, external view. Photo of Alexander E. Balakirev. **A** Dorsal view; **B** Ventral view; **C** Lateral view; **D** Belly, enlarged scale; **E** Head and backside, enlarged scale; **F** Distal half of tail with brush; **G** Hind foot, dorsal view.

The *Typhlomys* specimen recovered from Ta Xua (Fig. 2) exhibited a specific pattern of craniodental morphology, clearly distinct from that of the Lao Cai population referred to as *T.chapensis* studied previously (Abramov et al. 2012, Abramov et al. 2014). This applies in particular to the structure of the occlusive pattern of the molars and the general structure of the skull, which is discussed in detail below.

**Figure 3.**
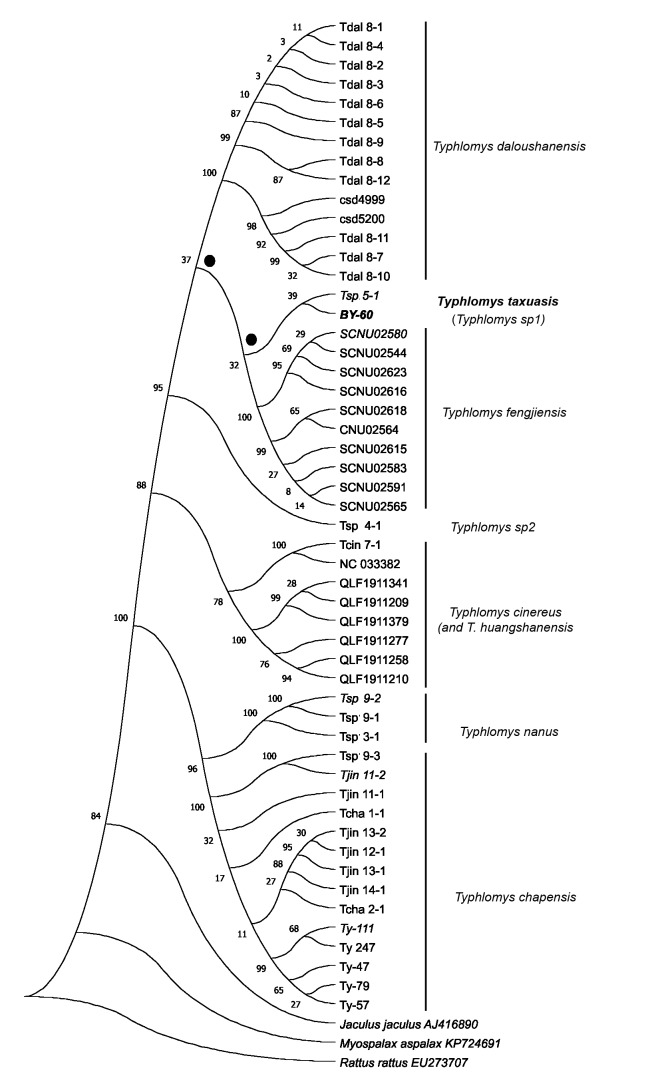
Phylogenetic tree (ML, *Cyt b*, *COI*, *GHR* concatenated dataset, GTR+G+I) for species and genetic lineages of *Typhlomys*. Bootstrap values over nodes; most ambiguous nodes are marked by black dotes.

The genetic analysis carried out allowed us to establish the level of its genetic uniqueness and its place in the overall diversity of *Typhlomys*. The phylogenetic tree constructed from the concatenated sequences of the three genes is shown in Fig. 3.

Our aims did not include constructing a detailed phylogeny and estimating divergence times; the analysis was performed for taxonomic purposes to accurately assign genetic attributions to the samples. As can be seen, the phylogenetic reconstruction data clearly indicate that the obtained sample belongs to the cinereus species group, while the characteristic genetic distances clearly reach the species level (Table 2) and are in the range of 0.105-0.196 (*Cyt b*, K2P). Genetic trees obtained for individual genes have the same topology, differing only in the level of support. Surprisingly, out of the established species of the genus, the closest relative is the recently described *T.fengjiensis*. It can also be seen that the closest related sequence available corresponds to one of the *Typhlomys* sp. 2 samples (Hu et al. 2021) from Mt. Laojun, Yunnan, China, N23.30 E103.95 (points 29 in Fig. 1). Their divergence level is 0.025 (K2P), so there is a reason to suppose that they belong to the same species.

## Data resources

The prepared skull and flat skin of the holotype are deposited in the Zoological Museum of Moscow State University, Moscow (ZMMU S-210284). Genetic data for new samples are deposited in GenBank under IDs PP987021-PP987022 and PP987159.

## Taxon treatments

### Typhlomys taxuansis sp. nov.

B35EB204-2034-5FFB-A16C-95F95F930450

B6350DEA-E691-45AC-A1EF-C8BC73327045

**Figure 4.**
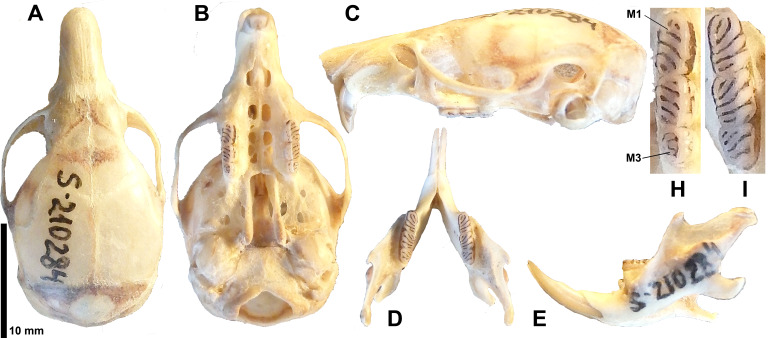
The holotype of *Typhlomystaxuansis*, skull, ZMMU S-210284 (field number: BY-60, adult female). **A** Dorsal view; **B** Ventral view; **C** Lateral view; **D** Lower jaw, dorsal view; **E** Lower jaw, lateral view; **H** M1-M3 upper molars, occlusal pattern, enlarged scale; **I** m1-m3 lower molars, occlusal pattern, enlarged scale.

**Holotype**: ZMMU S-210284 skull and flat skin (field number: BY-60), adult female (Figs. 2 and 4) collected on 25 November 2023, by Alexander E. Balakirev and Bui Xuan Phuong. The specimen deposited at the Zoological Museum of Moscow State University, Moscow, Russia, is shown in Fig. 4.

#### Materials

- **Type status:** Holotype. **Occurrence:** catalogNumber: ZMMU S-210284; recordNumber: BY-60; recordedBy: Alexander E. Balakirev; individualCount: 1; sex: female; lifeStage: adult; occurrenceStatus: present; preparations: skin; skull; disposition: in collection; associatedSequences: GenBank: PP987021-PP987022 and PP987159; occurrenceID: D570D565-0C0F-566C-8D44-DE2C3E91708F; **Taxon:** scientificName: *Typhlomystaxuansis*; **Location:** higherGeographyID: Ta Xua Nature Reserve, prov Son La, Vietnam; higherGeography: Asia; continent: Asia; country: Vietnam; countryCode: VN; stateProvince: Son La; county: Bac Yen; locality: 4 km east from Y Xoa Homestay; verbatimLocality: 4 km east from Y Xoa Homestay; minimumElevationInMeters: 2000; maximumElevationInMeters: 2200; decimalLatitude: 21.32218; decimalLongitude: 104.495873; geodeticDatum: WGS84

#### Description

**Measurements of holotype** (mm): BM = 21.10 g; HB = 85.0; TL = 117.0; HL = 25.0; EL = 18.0; ONL = 25.92; ZB = 13.85; IB = 5.59; LR = 8.34; BR = 3.67; BBC = 11.49; HBC = 8.55; ZBP = 1.78; LD = 7.08; LIF = 1.53; BIF = 1.87; LBP = 11.27; BBP = 4.00; PPL = 9.21; BMF = 1.78; LB = 3.42; CLM^_1-3_^ = 4.14; BM^1^ = 1.18; CLM_1-3_ = 4.29; and BM_1_ = 1.04.

One of the larger species within *Typhlomys* (HB = 85; ONL = 25.92). Vibrissae very long white; ears prominent, almost bare; eyes vestigial (Fig. 2). Dorsal body colouration: dark grey; entire ventral body from chin to anus, including inner side of limbs to wrists and knees; greyish due to dark grey hair base and white tip. Fingers four at fore-limbs and five on hind ones; hind feet slender and elongated (HL = 25 mm); plantar palms of all limbs light brown; fingers pale whitish; skin on dorsal surfaces of hind feet brownish, covered with slender hair. Tail long, well exceeding head and body length (TL = 117 mm), with scale rings; proximal third of the tail covered with extremely short and sparse hairs, back part is covered with longer hairs than the ventral side; and the distal half of the tail has tufts of long, dark grey hairs with no white inclusions.

The braincase is generally dome-shaped and relatively high due to its large size (HBC = 8.55 mm). The rostrum is straight beyond the upper incisors. Tympanic bullas are small. Zygomatic plate narrow; zygomatic arch strait, not incurvate on approximately equal thickness throughout its length. The incisive openings are small, rounded and have a pointed anterior edge. The bony palate is pierced by two pairs of additional symmetrical foramina, with the first pair, located under the rostrum, being approximately half as long as the posterior pair, located between the teeth. Diagonal bone trabeculae are clearly visible deep inside them. Dental formula is usual for the genus 1.0.0.3/1.0.0.3 = 16. M1, with almost equally wide anteroloph and posteroloph. M1 antherofosette is divided into two separate parts (Fig. 4H). The first molars of the upper jaws have six dark fossette-shaped structures; the first lower molars have only five dark fossette-shaped structures; the second upper and lower ones have four fosettes; and the third molars have two and four. The mesofossette on M1 is open on only the lingual sides. M2 with anterofossette, divided by a complete, well developed mesofosette; m1 with two antherofossettids and a closed mesofossettid. Anterofossettids are present in m2, but relatively short. m3 mesolophid has a crescent shape due to the facet protruding from the lingual side (Fig. 4H).

#### Diagnosis

The new species, morphologically, is most similar to *T.daloushanensis*, but can be distinguished, based on its dental and skull morphology. Based on genetic diversity, the most relative genetic lineage is *T.fengjiensis*. It obviously differs from geographically most adjacent species *T.chapensis* and *T.nanus* by a more flattened braincase; from all known *Typhlomys* species, except for *T.cinereus*, by zygomatic arch with deeper incurve; from *T.cinereus* by mesofossette on M1 open on both buccal and lingual sides rather than open on the buccal side only; and from *T.nanus* by posterofossettid on M1 present. The new species further differs from other species, except *T.daloushanensis*, by anterofossette on M2 present.

#### Etymology

The specific Latin name *taxuansis* composed as an adjective refers to its type locality in Ta Xua Nature Reserve, Son La Province, Bac Yen District, Vietnam. Due to the sampling location being the southernmost location currently known for this genus, we suggest “southern blind tree mouse” as the English common name.

#### Distribution

The new species is currently obtained only from the type locality, but may also be distributed in the adjacent mountainous areas in the northern-western part of Vietnam, north of the Da River. A close genetic similarity between the specimens and the Yunnan findings was found. Additionally, the genetic relationship with the *cinereus* group species, widespread in central and eastern China, suggests that the range of this species may extend eastwards to the provinces of north-eastern Vietnam, as well as the Chinese Province of Guanxi. Based on the ecological characteristics of habitats, it may also be distributed southwards, for example, at the Annamite Range, both on the Laotian and Vietnamese sides, but there are still no notes on its findings to the south from the Da River.

#### Ecology

The specimen investigated was captured in moist, misty mountainous forests at mid-altitudes (2000–2200 m a.s.l.). Sympatric species include *Neotetracussinensis*, *Eothenomysmiletus*, *Dremomysornatus*, *Dremomysgularis*, *Niviventerlotipes*, *Niviventerfulvescens*, *Muspahari* and *Leopoldamysedwardsi*, these being mostly the species of the Chinese mountain faunistic complex.

#### Conservation

The genus *Typhlomys* has recently been assessed for the IUCN Red List of Threatened Species in 2016. The only species recognised there to date, *Typhlomyscinereus*, is listed as Least Concern (Smith 2016). This is obviously outdated information and does not reflect current taxonomy improvements in this group. In fact, data on the conservation status of this rather exotic group of rodents is almost non-existent for most species. Here, we will try to close this gap to some extent, relying on modern data.

Based on literature sources and original data, we can compose a distribution map for the genus *Typhlomys* as shown in Fig. 1. The range area of the species, estimated from an ellipse containing all reliable finds, may range from about 1.27 mln km^2^ for *T.cinereus* with *T.huangshanensis*, 330000 km^2^ for *T.daloushanensis*, 60000–70000 for *T.chapensis* and *T.nanus*, about 12000 km^2^ for *T.taxuansis* to only 80–100 km^2^ for *T.fenjiensis*. At the same time, the real area of mountain forest within this zone is 10–20 times smaller. This circumstance is obviously of the utmost importance for environmental protection.

In agreement with IUCN rules (Anonymous 2001, Anonymous 2012), there are five quantitative criteria that are used to determine whether a taxon is threatened or not and, if threatened, to which category of threat it belongs (Critically Endangered, Endangered or Vulnerable). These five criteria are: population size reduction (past, present and/or projected); geographic range size and fragmentation, few locations, decline or fluctuations; small and declining population size and fragmentation, fluctuations or a few subpopulations; a very small population or very restricted distribution; and quantitative analysis of extinction risk.

Of the five circumstances given, only geographic range size and fragmentation match these species’ situation. The data currently available does not suggest either a very small population or any decline or reduction in population. For *T.cinereus* proper and *T.daloushanensis*, their natural ranges and the number of localities where the animals are listed allow for confirmation of their Least Concern (LC) status. For another, more profound inspection of needs. In agreement with IUCN rules, to qualify for criterion geographic range size and fragmentation, the general distributional threshold must first be met for one of the categories of threat, either in terms of extent of occurrence (EOO) or area of occupancy (AOO). The taxon must then meet at least two of the three options listed for this criterion. The options are: (a) severely fragmented or known to exist in no more than “X” locations; (b) continuing decline; or (c) extreme fluctuation (IUCN Anonymous 2001, IUCN Anonymous 2012). Of these criteria, only B1a is obviously applicable for all other species — fragmentation of the area due to the natural fragmentation of landscapes. It should be noticed that, within the category, *T.nanus*, *T.taxuansis* and *T.fengjiensis* could formally be classified as VU (Vulnerable) according to criterion B1 (Extent of Occurrence EOO) and, according to subcategory B2a (Area of Occupancy AOO). For *T.fengjiensis*, it is possible even to be Endangered due to the less than ten closely situated localities currently identified. However, there is still a little information about the natural situation to date.

For *T.taxuansis*, the range area of the species, estimated from an ellipse containing known finds, including closely-related Chinese samples, covers about 12,000 km^2^. At the same time, the real area of cloud forest vegetation within this zone is 10–20 times smaller. On the other hand, there is every reason to believe that the species is distributed much more widely, out of cloud forests and may well be in karst vegetation covering many hundreds of square kilometres in the regions of northern Indochina and southern China. It should also be noted that, in accordance with the IUCN rules, in the absence of any plausible threat to the taxon, the term "location" cannot be used and the subcriteria that refer to the number of locations will not be met. As far as is known to date, all the species apparently show neither a noticeable decrease in abundance (b) nor significant fluctuations (c) in relation to the size of the range or the number of individuals.

Thus, we believe that the category Least Concern may be applied to *T.cinereus* and *T.daloushanensis*, along with Near Threatened B1a+2a and that the current population trend is stable for *T.chapensis*, *T.nanus*, *T.taxuansis* and *T.fengjiensis* species. The main threats to its conservation are not primarily linked to direct impacts and population reduction, but rather to its association with specific and highly- specialised habitat types, such as high-level mountain and cloud forest vegetation, which may limit the potential distribution for many species.

#### Taxon discussion

The molecular dating analyses suggested that divergences within *Typhlomys* started during the middle or late Miocene. Divergence in the Miocene is usually considered a genus-level diversification (Jansa et al. 2009). In China, remains of several species of blind tree mice have been found in the Upper Miocene (Qiu 1989, Qiu and Ni 2019), Pliopleistocene (Qiu Z and Jin C-Z 2017), Lower Pleistocene (Zheng 1993,Fejfar and Kalthoff 1999, Zheng 2004, Jin et al. 2009, Wang et al. 2014) and lower Upper Pleistocene. This timing is congruent with fossil records of Platacanthomyidae (Qiu 1989). A special pattern of tooth morphology was also found in four congeneric fossil species from the late Miocene to the Pleistocene in China (Qiu 1989, Zheng 1993). However, the recent species available only differed from each other by appearance, size and tender features of pelage colour. The patterns and structures of their molar teeth for many species are rather similar. Considerable differences in tooth size and tooth crown height were observed in fossil species, but the patterns and structures of teeth have remained stable since the early Pleistocene. However, as we can see, in a number of cases, quite distinct morphological features of the structure of the chewing surface can be detected, marking the species.

The palaeontological history of *Typhlomys* in Vietnam has been largely unknown until recently, but this year a fossil of *Typhlomysstegodontis* sp. nov. was described, based on a maxillary fragment and isolated teeth from the Middle Pleistocene Tham Hai cave locality in northern Vietnam (Lang Son Province, about 200 km NE from Ta Xua). This first finding of the fossil Platacanthomyidae in Vietnam fills the Middle Pleistocene gap in the palaeontological record of the family (Lopatin 2024). Interestingly, the parafossette M1 in the holotype of the fossil *T.stegodontis* is obviously bifurcated, with an anterolingual branch reaching the enamel wall of the tooth. This structure is largely similar to the completely isolated additional fossette region observed in M1 *T.taxuansis*, constituting one of its unique features. This similarity, as well as the geographical location of the finding, suggests an evolutionary relationship between these taxa. Unfortunately, the third upper molar of the fossil species has not yet been discovered, but surveys continue.

The distribution of the different *Typhlomys* species demonstrates a distinct geographic pattern, which could be partly due to the complex topography and low dispersal ability of many animals (Fu and Zeng 2008, Zhou et al. 2012, Cheng et al. 2017, Hinckley et al. 2020). On the other hand, in Yunnan, scattered mountain ranges with elevations over 3000 m., such as the Wuyi and Huangshan Mountains, form patches of “sky islands” favoured allopatry in isolated areas (McCormack et al. 2009). It should be noted that, taking into account new data, these islands of high-mountain habitats are occupied by representatives of the chapensis group and the cinereus group, which are mainly distributed in the east and often occupy lower-lying habitats. In addition, mountains provide a wide altitudinal range that helps buffer climate changes and has provided initially continuously suitable habitats for *Typhlomys* since the early Late Miocene (Wu et al. 2013, He et al. 2019). This may indicate that, during the Late Miocene, when the most rapid speciation within the genus *Typhlomys* occurred, this taxon may have been a less pronounced montane group. Some of the originally temperate montane populations could have been locked within a lower montane region and were forced to adapt to an increasingly high-altitude climate as the mountains rose and montane vegetation belts moved in the Pleistocene. This may explain the proximity of ranges with true allopatry of *T.nanus*, *T.cheapens*, *T.taxuansis* and several as yet undescribed species and forms in the mountains of Yunnan and northern Vietnam. Lately, the complex topography of Yunnan mountains may facilitate allopatric speciation by physical isolation, eventually resulting in a series of endemic species with narrow isolated areas. Up till now, there are still a number of populations without genetic attribution from many regions (Pu et al. 2022). Thus, the number of species of the genus *Typhlomys* in China and Vietnam still requires further profound biodiversity surveys, taxonomic and phylogenetic studies, which may result in many interesting findings.

## Identification Keys

### Key to the established species of *Typhlomys*

**Table:** 

1	Braincase flattened, molars wide, anterofossette on M1 relatively wide, mesofossettid on m1 closed	2
–	Braincase dome-shaped, molars narrow, anterofossette on M1 narrow, mesofossettid on m1 open buccally	4
2	ONL < 23.0 mm, LD < 11.0 mm, dorsal surface of hind feet covered with blackish hairs	T.cinereus*
–	ONL > 23.7 mm, LD > 11.4 mm, hind feet dorsal surface yellowish-white or dark	3
3	ONL > 23.7 mm, LD > 11.4 mm, hind feet dorsal surface yellowish	*T.daloushanensis*
–	ONL even larger, 25.38 ± 0.77 mm, Zygomatic arch incurved at posterior two-fifths, anterior part narrower than posterior part. Dorsal surfaces of hind feet brown, covered with dark hair	*T.fengjiensis*
4	ONL > 21.6 mm, LD > 10.6 mm, IB > 5.0 mm, posterofossettid and posterolophid on m1 present	5
–	ONL < 22.2 mm, LD < 10.2 mm, IB < 4.9 mm, posterofossettid absent on m1, anterolingual end of m2 fillet-shaped	*T.nanus*
5	There are three linear parallel fossettes on m3, m1 antherofosette single, linear	T.chapensis**
–	m3 mesolophid has a crescent shape due to the facet protruding from the lingual side, so there are only two fossettes present. m1 antherofosette is divided into two separate parts. Dorsal surfaces of hind feet are brown; the tail brush is without whitish hairs.	*T.taxuansis*

Notes:* *T.cinereus* includes *T.c.jingdongensis* as a younger synonym. **: *T.chapensis* includes *T.c.guangxiensis* as a younger synonym.

## Supplementary Material

XML Treatment for Typhlomys taxuansis

## Figures and Tables

**Table 1.** Geographical locations for *Typhlomys* sp samples are available (only geographically attributed samples are listed).

#	Site Sample locality	Latitude (N)	Longitude (E)	Elevation m	Species	Voucher specimen or tissue sample	Citation
1	Mt. Qinling, Zhashui County of Shangluo, Shaanxi, China	33.8	109.2	1300	*Typhlomysdaloushanensis*		(Wu 1990)
2	Maozhai Nature Reserve, Sichuan, China	32.85596	10.47216	1070	*Typhlomysdaloushanensis*		(Liu et al. 2007)
3	Mt. Guangwushan, Sichuan, China	32.57	106.74		*Typhlomysdaloushanensis*	GWS20210425001	(Pu et al. 2022)
4	Shennongjia forestry region of Hubei，China	31.54	110.40	2000-2300	*Typhlomysdaloushanensis*		(Liu and Wang 1997)
5	Kaixian, Chongqing, China	31.28	108.35		*Typhlomysdaloushanensis*	SAF16709	(Pu et al. 2022)
6	Fenjie, Chongqing, China	30.712946	109.562642	1880	*Typhlomysfengjiensis*	SCNU02580; SCNU02583	(Pu et al. 2022)
7	Fenjie, Chongqing, China	30.662742	109.520595	1883	*Typhlomysfengjiensis*	SCNU02591; SCNU02616	(Pu et al. 2022)
8	Shiruguan in Xinglong Town, Fengjie County, Chongqing, China	30.648639	109.489543	1579	*Typhlomysfengjiensis*	SCNU02544, SCNU02564, SCNU02565 SAF97118, SAF97119	(Pu et al. 2022)
9	Fenjie, Chongqing, China	30.645056	109.492861	1827	*Typhlomysfengjiensis*	SCNU02616; SCNU02615	(Pu et al. 2022)
10	Fenjie, Chongqing, China	30.644861	109.491816	1857	*Typhlomysfengjiensis*	SCNU02564; SCNU02565; SCNU02618; SCNU02623	(Pu et al. 2022)
11	Mt, Xingdou, Hubei, China	30.217	108.724		*Typhlomysdaloushanensis*		(Pu et al. 2022)
12	Mt.Tianmu, Zhejiang, China	30.21	119.5	1100-2300	*Typhlomyscinereus*		(Zhuge et al. 1985)
13	Mt. Huangshan, Anhui, China	30.119	118.306	710	*Typhlomyshuangshanensis*	AE1901HS01; AE1902HS02; AE1902HS03	(Hu et al. 2021)
14	Badagongshan National Nature Reserve, Hunan, China	29.65	109.83		*Typhlomyscinereus*		(Xie et al. 2014)
15	Mt. Jinfo, Chongqing, China	29.01867	107.1905	2105	*Typhlomysdaloushanensis*	KIZ033551; KIZ033589; KIZ033590; KIZ033594; KIZ033595; KIZ033596	(Cheng et al. 2017)
16	Mt. Jinfo, Chongqing, China	29.00353	107.1883	1997	*Typhlomysdaloushanensis*	KIZ033599; KIZ033600; KIZ033555; KIZ033555; KIZ033556	(Cheng et al. 2017)
17	Mt. Jinfo, Chongqing, China	29.00178	107.1891	1997	*Typhlomysdaloushanensis*	KIZ033552	(Cheng et al. 2017)
18	Kuankuoshui of Suiyang County, Guizhou, China	27.97	107.25	1600	*Typhlomysdaloushanensis*		(Wang et al. 1996)
19	Mt. Wuyi, Fujian, China	27.63675	117.9041111	400	*Typhlomyscinereus*	USNM238223; KIZ:Z201312257	(Cheng et al. 2017)
20	Mt. Jiaozi, Yunnan, China	26.08277778	102.84675	3252	*Typhlomysnanus*	KIZ033585	(Cheng et al. 2017)
21	Mt. Jiaozi, Yunnan, China	26.06661111	102.8254444	3204	*Typhlomysnanus*	KIZ033584; KIZ:033586	(Cheng et al. 2017)
22	Libo County of Qiannan, Guizhou, China	25.35	107.92	747	*Typhlomys* sp. 1	ROM118593	(Cheng et al. 2017), (Su et al. 2020)
23	Mt. Ailao, Yunnan, China	24.57	101.48	2572	*Typhlomyschapensis*	KIZ033591	(Cheng et al. 2017)
24	Mt. Ailao, Yunnan, China	24.5	101.4	2791	*Typhlomyschapensis*	KIZ031851; KIZ029295	(Cheng et al. 2017)
25	Mt. Wuliang, Yunnan, China	24.35	100.7333333		*Typhlomyschapensis*	KIZ019150; KIZ019152	(Cheng et al. 2017)
26	Mt. Ailao, Yunnan, China	24.28419444	101.2603889	2380	*Typhlomyschapensis*	KIZ033589	(Cheng et al. 2017)
27	Mt.Daming, Guangxi, China	23.75	108.517	1775	*Typhlomyscinereus*		(Liang 1980)
28	Binlin, Guangxi, China	23.5	108.5		*Typhlomyscinereus*		(Wang et al. 1996)
29	Mt. Laojun, Yunnan, China	23.3	103.95	2043	*Typhlomys* sp. 2	1503001	(Cheng et al. 2017)
30	Mt. Dawei, Yunnan, China	23.03661111	103.5271667	2013	*Typhlomys* sp. 2	1112103	(Cheng et al. 2017)
31	Mt. Dawei, Yunnan, China	22.91330556	103.6977222	2038	*Typhlomysnanus*	KIZ:028335; KIZ:028336	(Cheng et al. 2017)
32	Mt. Huanglian, Yunnan, China	22.87033333	103.2355556	2232	*Typhlomyschapensis*	KIZ033588	(Cheng et al. 2017)
33	Mt, Huanglian, Yunnan, China	22.87	102.28	1955	*Typhlomyschapensis*	KIZ033587	(Cheng et al. 2017)
34	Mt, Huanglian, Yunnan, China	22.87	103.24	2232	*Typhlomyschapensis*	KIZ:033588	(Cheng et al. 2017)
35	Mt. Huanglian, Yunnan, China	22.867	102.2838333	1955	*Typhlomyschapensis*	KIZ033587	(Cheng et al. 2017)
36	Mt. Phan Xi Pang, Lao Cai, Vietnam	22.35	103.77	1926	*Typhlomyschapensis*	ZIN101563; ZIN1015634; ZIN1015635; ZIN1015636; ZIN101567; ZIN99914; ZIN99916; ZIN100882; ZIN100883; ZIN100411	(Abramov et al. 2014), (Cheng et al. 2017)
37	Ta Xua Nature Reserve, Son La, Vietnam	21.32218	104.495873	2100	*Typhlomystaxuansis*	ZMMU S-210284	new original data

**Table 2.** Estimates of evolutionary divergence within *Typhlomys* species as accessed by the *Cyt b* gene (Nei and Kumar 2000). Standard error estimates are shown above the diagonal. Analyses were conducted using the Maximum Composite Likelihood model (Tamura et al. 2004).

	Intergroup divergence								Intragroup divergence		
	*T.daloushanensis*	*T.chapensis*	*T.cinereus*	*T.fengjiensis*	*T.nanus*	*T.* sp. 1	*T.* sp. 2 (4-1)	*T.taxuansis*			
*T.daloushanensis*		0.0177	0.0159	0.0127	0.0186	0.0180	0.0142	0.0141	*T.daloushanensis*	0.0188	0.0030
*T.chapensis*	0.2031		0.0165	0.0176	0.0131	0.0039	0. 0168	0.0202	*T.chapensis*	0.0117	0.0024
*T.cinereus*	0.1967	0.2127		0.0136	0.0163	0.0172	0.0148	0.0160	*T.cinereus*	0.0970	0.0089
*T.fengjiensis*	0.1263	0.1947	0.1666		0.0180	0.0179	0.0128	0.0118	*T.fengjiensis*	0.0080	0.0019
*T.nanus*	0.2130	0.1410	0.2086	0.2044		0.0129	0.0171	0.0198	*T.nanus*	0.0466	0.0063
*T.* sp. 1	0.1971	0.0193	0.2086	0.1874	0.1322		0.0175	0.0200	*T.* sp. 1	0.0000	0.0000
*T.* sp. 2 (voucher 4-1)	0.1335	0.1759	0.1729	0.1099	0.1870	0.1768		0.0138	*T.* sp. 2 (voucher 4-1)	n/c	n/c
*T.taxuansis*	0.1290	0.2007	0.1775	0.1050	0.2037	0.1906	0,1179		*T.taxuansis*	0.0934	0.0165
